# HIV/AIDS communication in four Nigerian mainstream newspapers

**DOI:** 10.11694/pamj.supp.2014.17.1.3672

**Published:** 2014-01-18

**Authors:** Onjefu Okidu

**Affiliations:** 1 Department of Mass Communication, Caleb University, Lagos, Nigeria

**Keywords:** Cognitive, activity, low and middle income countries, mass media, context, newspaper, agenda, HIV/AIDS communication

## Abstract

**Introduction:**

One consensus in discussions on HIV/AIDS communication in low and middle income countries (LMICs) is the need for communication models to focus on activity rather than cognitive indicators in order to achieve desired improvements in health behaviors and outcomes. Past failures of HIV/AIDS communication efforts in LMICs have been attributed to emphasis on cognitive indicators. This study analyses HIV/AIDS communication models in Nigerian newspapers.

**Methods:**

Data were obtained through analysis of manifest content of four Nigerian papers issued between 2002 and 2004. Frequency, prominence and space dedicated to HIV/AIDS-related topics were measured. Descriptive statistics were used to highlight the frequency and percentage of cognitive- and activity-oriented informationon HIV/AIDS.

**Results:**

A total of 464 HIV/AIDS-related articles were identified. Fifty-nine percent (274) of articles were activity-oriented. Over half of articles were news stories. No news story made front and back pages lead. There were only nine editorials on HIV/AIDS.

**Conclusion:**

This study shows that the activity model of HIV/AIDS communication dominates the four Nigerian mainstream newspapers studied. However, it is worth noting the limited number of editorials and feature articles, which have the capacity to stimulate debate and foster a social environment in which AIDS is addressed in a spirit of openness. For a country that has thethird largest number of people living with HIV/AIDS globally, one would expect the mass media to deliberately play an instrumental and a more active role in the battle against the disease by engaging in in-depth contextual discourse on HIV/AIDS.

## Introduction

Current thought in HIV/AIDS communication in low and middle income countries foregrounds the need to move beyond the cognitive approach to the activity approach [1].Cognitive models of behaviour change communication like the Health Belief Model, the Theory of Reasoned Action, Social Learning Theory, and the AIDs Risk Reduction Model (ARRM), which dominated the first decade of social science research on HIV/AIDS, assume that individual reason provides the impetus for human action [2].The models seek to interpret and analyze health behaviours at the individual level. HIV/AIDS cognitive information refers to information about HIV/AIDS that focuses on individual self-efficacy and emphasizes the simple, linear relationship between individual knowledge and action [3].The activity model of behaviour change communication derives from the activity theory. This model views behavioral outcome as a product of the individual's context and argues that human activity is complex and socially-bound and driven and not simply the sum of individual actions [4]. HIV/AIDS activity information was defined as information about HIV/AIDS that extends the framing of HIV/AIDS from primarily an individual function to one that is linked to more macro socio-economic, cultural and political contexts [5].

Although, cognitive models have been effective in western contexts [6], some scholars and practitioners argue that these models are inadequate for HIV/AIDS communication in low and middle income countries [6]. Following this realization, calls have been made for a shift from the cognitive to the activity model of HIV/AIDS communication [6]. The importance of a nationally-driven agenda in lowering incidence and mitigating the impact of HIV/AIDS in low and middle income countries was not lost on Nigeria which, in 2001,launched a multi-sectoral and community-based response to the epidemic, exemplified in the HIV/AIDS Emergency Action Plan (HEAP;2001-2004) and the National Strategic Framework (NSF; 2005-2009).

However, a dominant view in the literature is that the mass media in low and middle income countries are yet to transition from cognitive models to the activity model of HIV communication [6]. Presently, there is little literature systematically addressing the response of the mass media to HIV/AIDS in low and middle income countries. The severity of the HIV epidemic in low and middle income countries and the immense potential that the media have to contribute to its prevention and control both justify the need for an examination of their media content in order to ascertain the level of response. Such an examination may yield information that can help strengthen policies and advance effective strategic partnerships in HIV/AIDS prevention efforts.

## Methods

Using data from a recent comparative study on HIV/AIDS cognitive and activity-oriented information content in four mainstream Nigerian newspapers, this paper examines; (a) the number of HIV/AIDS cognitive and activity information carried by Nigerian newspapers (b) amount of space devoted to HIV/AIDS cognitive and activity information by Nigerian newspapers, and (c) the location of HIV/AIDS cognitive and activity information in Nigerian newspapers. In other words, three variables (frequency, space and prominence) were measured in this study which covered a three-year period (2002– 2004).

Although HIV was first reported in Nigeria in 1986, the study period coincided with the implementation of the first phase of Nigeria'smulti-sectoral and community-based national response to the epidemic. The four newspapers surveyed in the present study are the Guardian, Punch, New Nigerian and Daily Trust. The Guardian and Punch are published in southern Nigeria while New Nigerian and Daily Trust are published in the North. These papers represent an important outlet for health information. They have been in the forefront of the crusade against HIV/AIDS [7]. In addition, newspapers are widely cited sources of information on HIV/AIDS issues in Nigeria [8].

Coding: Coders, working in pairs, coded newspaper items independently of one another. Each pair of coders coded all issues of the same newspaper during the study period according to the coding scheme. Coding focused on manifest content. Data extraction and recording were performed manually. All coders were final-year undergraduate students of Mass Communication enrolled in print journalism in three Nigerian tertiary institutions (the University of Lagos, Lagos State University and Kaduna Polytechnic). The coders were carefully recruited and trained to code. The training of coders included one 2-hour session a day for two weeks. The categories for coding HIV/AIDS-related content of the newspapers were based on those developed by Journalists Against AIDS (JAAIDS), Nigeria in 2003 on the coverage of HIV/AIDS in eleven Nigerian newspapers. The JAAIDS categories are similar to those Pratt et al [9] used in their study of HIV/AIDS information in African popular magazines and medical journals. According to Stempel [10], “there are real advantages to using a category system that has been used in other studies” because validity and reliability are largely addressed.

Intercoder Reliability: Intercoder reliability was measured using Krippendorff's alpha [11]. Krippendorff's alpha score for the Newspapers ranged from 0.891 to 0.944 (The Guardian, 0.891; The Punch, 0.918; New Nigerian, 0.936; Trust, 0.944). Considering these results, the coding sheet and the coders were deemed fit for the study.

### Analysis

The units of analysis were all newspaper items (news stories, feature articles, editorials, opinions, letters to the editor, photos, cartoons, advertisements etc.). Each unit of analysis was coded as cognitive- or activity-focused based on the following categories: (1)Awareness/Prevention, (2)Treatment/Care, (3)Advocacy and campaign, (4)Cure claims, (5)Policy pronouncements, (6)Litigation, (7)Statistics/Trends, and (8)Research. As such, the frequency of either HIV/AIDS cognitive or activity information was measured by the number of items about HIV/AIDS in Nigerian newspapers. In terms of space, the items were measured in column centimeters (col. cms). The study quantified how much space is given to HIV/AIDS cognitive and activity information by multiplying the number of published HIV/AIDS items’ columns in the selected newspapers by their length in centimeters. The centimeters were measured by placing vertically a centimeter ruler alongside columns of published HIV/AIDS items.

A modification of Budd's [12] attention scores technique, a device for measuring news play and usually applied in print media content analysis, was employed in measuring newspaper items prominence. Rather than its limited application to HIV/AIDS news stories which the researcher defined as news stories with an HIV/AIDS theme, the technique was applied to HIV/AIDS information. HIV/AIDS information was defined as all newspaper items (news stories, features, editorials, advertisements etc.) about or relating to HIV/AIDS. Consequently, (1) when an information item in any of the content categories constituted a front page lead, six points (attention scorers) were allotted to that item's content category;(2)When an information item constituted a back page lead, five points (attention scores) were allotted to that item's content category; (3)When an information item appeared on the front page, four points (attention scores) were allotted to that item's content category; (4) When an information item appeared on the back page, three points (attention scores) were allotted to that content category; (5) When an information item appeared on page two, two points (attention scores) were allotted to that item's content category; and (6) When an information item appeared on other inside pages, 1 point (attention score) was allotted to that item content category.

It is assumed that the most important items are published on the front page with the lead outstanding, while the degree of priority on information items decreases into the inside pages. The back page is also considered critical and strategic because of its external position. For the study therefore, information items which were not published on front and back pages of the newspapers were considered of least attention or priority. Items that continued from the two pages (front and back) to inside pages have been classified as front and back (page).

## Results

Coders identified a total of 464 pieces of HIV/AIDS-related information in the four newspapers corresponding to a total attention score of 571 and 22293.8 column centimeters of information space. The data were analyzed to ascertain the attention paid to HIV/AIDS cognitive-oriented information and HIV/AIDS activity-oriented information in terms of information frequency, prominence and space. One hundred and forty five (31%) pieces of HIV/AIDS-related information were in The Punch; 116 (25%) in the New Nigerian; 111 (24%) in The Guardian, and 92 (20%) in the Daily Trust. In terms of prominence, The Punch had the highest attention scores, 176 (31%). Daily Trust had 150 (26%); New Nigerian, 131 (23%) and The Guardian, 114 (20%). New Nigerian recorded the highest column centimeters with regards to HIV/AIDS information space, 6771.9 (30%); The Punch, 6023.6 (27%); Daily Trust, 5263.5 (24%) and The Guardian, 4234.8 (19%).The total number of HIV/AIDS activity-oriented information published by the four Nigerian newspapers was more than the total number of HIV/AIDS cognitive-oriented information during the period studied. Two hundred and seventy four (59%) pieces of HIV/AIDS-related information were activity-oriented. Apart from the Daily Trust which published an equal number of activity and cognitive information, all the other newspapers published more activity than cognitive information. The proportions of activity-oriented pieces of HIV/AIDS-related information were 64.7% in the New Nigerian; 61.4% in The Punch; 57.7% in The Guardian and 50% in the Daily Trust.

Altogether, the four newspapers devoted a total of 276 news stories to HIV/AIDS (64.9% activity-oriented). The proportions of activity-oriented HIV/AIDS news stories in the Daily Trust was 59.3%; The Guardian 61.7%; New Nigerian, 67.7%, and The Punch 67.1%. Out of the 279 attention scores allotted to all the news stories in the content categories, activity-oriented news stories had more points than cognitive-oriented news stories; 70.6% (197). No news story on HIV/AIDS made front and back pages lead. There were equal numbers of HIV/AIDS activity and cognitive front page news stories. However, among the news stories which constituted the back page, page 2 and other inside pages, activity-oriented news stories had more attention scores than cognitive-oriented news stories. Overall, HIV/AIDS news stories occupied 11637.8 column centimeters. While HIV/AIDS activity-oriented news stories accounted for 59.7% (6951.9col.cms), HIV/AIDS cognitive-oriented news stories accounted for 40.3% (or 4685.9 col.cms).

Two of the four newspapers (Daily Trust and New Nigerian) did not publish HIV/AIDS editorials at all during the period studied. There were nine editorials on HIV/AIDS. Eight of these appeared in The Guardian. Of the eight editorials in The Guardian, five were activity-oriented. The only editorial featured in The Punch was cognitive-oriented.

There were more HIV/AIDS activity-oriented feature articles (60.7%) than HIV/AIDS cognitive-oriented feature articles. Seventy-one percent of feature articles in the Daily Trust were activity oriented as were 56.4% in The Guardian, 60.0% in the New Nigerian, and 63.6% in the Punch. The ratio of space in col.cms occupied by HIV/AIDS activity-oriented feature articles to HIV/AIDS cognitive-oriented feature articles in all the newspapers put together indicates that HIV/AIDS activity-oriented feature articles occupied more space than HIV/AIDS cognitive feature articles. During the period studied, the four Nigerian newspapers devoted a space of 5341.1 col.cms (or 50.6%) to activity-oriented feature articles and a space of 5221 col.cms (or 49.4%) to cognitive-oriented feature articles. In the New Nigerian and the Punch however, HIV/AIDS cognitive-oriented feature articles occupied more space than HIV/AIDS activity-oriented feature articles. In New Nigerian 44.4% (240col.cms) of space was dedicated to activity-oriented feature articles while 48.6% (2040 col.cms) was dedicated to activity-oriented feature stories. The Guardian and the Daily Trustdevoted more space to HIV/AIDS activity oriented feature articles (56.4% (2640.5 col.cms) and 70% (420.6 col.cms) respectively.

There were thirty-five HIV-AIDS-related advertisements in the four newspapers. Overall, there were more cognitive-oriented advertisements (60.0%). However, a greater proportion of advertisement messages in The Guardian and New Nigerian had three and two HIV/AIDS-related advertisements respectively, all of which were activity-oriented. The Daily Trust had 21 advertisements of which 16 (76.2%) were cognitive-oriented. There were nine HIV/AIDS-related advertisements in The Punch of which five were cognitive-oriented.

## Discussion

To understand the response of the media in Nigeria to the call for transition from cognitive to activity-oriented models of HIV/AIDS communication, a comparative analysis of HIV/AIDS information in four Nigerian mainstream newspapers was conducted. The predominance in coverage of HIV/AIDS activity-oriented information as identified in the four newspapers suggests that HIV/AIDS contextual issues receive considerable attention. The dominance of activity-oriented HIV/AIDS content information contradicts the view of some scholars [1, 5, 13–16] that insufficient attention has been paid by the mass media in developing countries to the activity model of HIV/AIDS communication. It also suggests that Nigerian newspapers might have been responding to global reviews of HIV/AIDS communication, such as, for example, the new UNAIDS (1999) communication framework. The UNAIDS framework calls for a shift from individual-level theories and models of preventive health behaviours (health belief models, theory of reasoned action, stages of change, AIDS Risk Reduction Model and others) to multilevel models that take into account cultural and contextual factors.

The dominance of activity information further shows that Nigerian newspapers in their expanded roles may be capable of providing national and international models of strategic responses to diseases. The dominance of HIV/AIDS activity-oriented editorials as well as feature articles is critical because these items, more than news stories, have the capacity to lay bare the ways in which HIV/AIDS exacerbates social prejudices, economic inequalities, discriminatory practices and political injustices. Although, editorials and feature articles have the capacity to stimulate debate and foster a social environment in which AIDS is addressed in a spirit of openness, news stories dominated the HIV/AIDS information items studied. The relative lack of editorials and feature articles may suggest ambivalence occasioned by lack of skill and acknowledge on the part of editorial and feature writers.

None of the papers sampled used an HIV/AIDS story as front or back page lead. Furthermore, both HIV/AIDS activity and cognitive stories have very low attention scores on the front and back pages. A number of reasons could be adduced for it. It could be that the urge to sell had conditioned the perception of the newspapers towards considering HIV/AIDS stories less sensational than other news items. It could also be a manifestation of the natural inclination of the papers to follow occurrences that had “greater” social impact. Indeed, the Nigerian mass media within the period covered by this study focused more on intriguing and sensational social, political and economic issues in Nigeria.

A striking feature of the four Nigerian newspapers coverage of HIV/AIDS information was the dominance of HIV/AIDS cognitive-oriented advertisement messages. This is not surprising, as advertisers and advertisement copy writers in Nigeria as elsewhere determine the content and space of advertisement messages.

Study findings should be interpreted in light of several limitations. First, the sample was not randomized and is based on a small sample of newspapers. Results cannot therefore be generalized to the entire Nigerian newspapers and other mass media. Second, tracing the evolution of newspaper reporting on HIV in Nigeria over a longer period of time would have offered a broader comparative analysis of the transition from cognitive to activity model of communication. These are areas for further research.

## Conclusion

This study shows that the activity model of HIV/AIDS communication dominates the four Nigerian mainstream newspapers studied. It is possible that this trend is common to other Nigerian mass media outlets. Perhaps, the considerable global review of HIV/AIDS communication practices that have taken place and the relative positive national response have provided broader basis for contextual redirection for the mass media in some developing countries. However, it is worth noting the limited number of editorials and feature articles, which have the capacity to stimulate debate and foster a social environment in which AIDS is addressed in a spirit of openness.For a country that has the third largest number of people living with HIV/AIDS, globally, one would expect the mass media to deliberately play an instrumental and a more active role in the battle against the disease by engaging in more in-depth contextual discourse.
